# A New Pearl in Chronic Venous Disease Pathophysiology—The Duplex Ultrasound and the Elastographic Features of Lymph Nodes Varicose Veins in the Groin

**DOI:** 10.3390/diagnostics16060905

**Published:** 2026-03-18

**Authors:** Ioana-Teofana Dulgheriu, Carolina Solomon, Stefan Timofte, Anca-Ileana Ciurea, Sorin Marian Dudea

**Affiliations:** 1 Department of Radiology and Medical Imaging, Faculty of Medicine, “Iuliu Hatieganu” University of Medicine and Pharmacy, 400012 Cluj-Napoca, Romania; 2 Department of Radiology, Emergency Clinical County Hospital Cluj-Napoca, 400006 Cluj-Napoca, Romania; 3 Department of Cardiology, “Prof. Th. Burghele” Clinical Hospital, 050653 Bucharest, Romania

**Keywords:** Doppler ultrasound, chronic venous disease, lymph node venous network

## Abstract

**Background/Objectives**: Chronic venous disease (CVD) is a prevalent condition marked by valve dysfunction and increased pressure in lower limb veins. The trans-nodal veins in the inguinal region and Scarpa triangle, which connect the superficial and deep venous systems, provide new insight into venous insufficiency pathways. While they function normally in healthy individuals, they can become dilated in chronic venous disease or following surgery. The purpose of this study was to provide an ultrasonographic anatomical description of intranodal varicose veins and to assess possible changes in the stiffness of varicose, dilated inguinal lymph nodes. **Methods**: The study comprised 92 participants, including 69 women and 23 men, who underwent Doppler ultrasound examinations of the lower-limb venous system, focusing on the groin from both a descriptive morphological and an elastographic perspective. The diagnosis of lymph node varices was made according to established criteria, its severity was assessed using an original classification system, and shear-wave elastography (SWE) values were recorded. **Results**: More than 83% of patients with operated CVD had lymph node varicose veins. Patients with lymph node varicose veins had larger groin lymph node diameters than patients with CVD without lymph node varicose pathology. The mean shear wave elastography values were significantly lower in the group with lymph node varices compared to the group without (12.2 ± 1.1 kPa vs. 20.1 ± 2.3 kPa; *p* < 0.05). Elastographic values correlate with lymph node diameter (*p* = 0.039) and with varicose vein grade (*p* < 0.001). **Conclusions**: Intranodal varices may indicate disease progression. These vascular abnormalities impact SWE measurements by altering tissue mechanics. It is imperative to consider the interactions between the lymphatic and venous systems in the management of CVD to improve patient outcomes.

## 1. Introduction

Chronic venous disease (CVD) represents a frequent and debilitating medical condition characterized by various pathological alterations, from lower limb oedema to pain and changes in skin integrity. It is prevalent worldwide and significantly affects patients’ quality of life [1]. If not addressed, it progresses to a great spectrum of complications such as postphlebitic syndrome and intractable venous ulcers [2,3,4]. The diagnosis of CVD is based on clinical features and complementary imaging techniques. An international consensus conference developed the Clinical, Etiology, Anatomic, and Pathophysiology (CEAP) classification to improve consistency in reporting, diagnosis, and management of CVD [5]. Venous duplex ultrasound is the primary modality for confirming a diagnosis of chronic venous insufficiency (CVI) and is considered the gold standard [6]. Contributing factors to CVD, apart from genetic factors, have been considered female sex, advanced age, prolonged standing [7], and high body mass index (BMI).

The underlying mechanisms of venous insufficiency have been extensively studied. Valve impairment and chronic venous hypertension trigger the release of inflammatory mediators, which cause incipient structural changes, such as an imbalance between collagen and elastin [8,9,10]. However, certain pathways remain underexplored from a pathophysiologic perspective, and our understanding of their potential effects on postoperative relapses remains limited. Due to insufficient exploration of the descriptive (morphologic) and functional nature of these pathways, treatment options remain unclear. Such a pathway is the lymph node venous network (LNVN) in the groin. It represents trans-nodal veins in the inguinal region and the Scarpa triangle, connecting the superficial and deep venous systems, as described in normal subjects [11] and in pathologically dilated post-surgical cases or non-operated CVI [12,13,14,15,16]. This study aimed to provide an ultrasonographic anatomical description of intranodal varicose veins, to analyze the correlation between the magnitude of intranodal vein dilation and clinical changes, as well as potential changes in lymph node elasticity. A secondary objective of the study was to assess the association between common diseases (e.g., arterial hypertension and diabetes) and the CEAP clinical stage.

## 2. Materials and Methods

A prospective observational study was conducted in the Vascular Ultrasound Division of the Radiology Department at a County Clinical Emergency Hospital between January 2024 and September 2025. All patients provided informed consent and were examined by the same consultant radiologist, who had 6 years’ experience in vascular sonography.

For each patient, the following clinical information was recorded: age, gender, BMI, urban/rural residence, clinical C grading (with the severity grading of the skin changes, according to the CEAP classification [5]), family history, previous surgery for CVD, and the presence of other diseases (such as arterial hypertension and diabetes mellitus).

### 2.1. Inclusion Criteria

Participants were adult patients referred for CVD work-up from the Dermatology, Vascular, and General Surgery Departments.

### 2.2. Exclusion Criteria

Pregnant or breastfeeding women were excluded from the study. Other exclusion criteria included patients with lymphedema or systemic edema, systemic diseases (e.g., history of tuberculosis, lymphoma, sarcoidosis), and local lower-limb infections.

### 2.3. Ultrasound Examination

All participants were examined using a Philips EPIQ Elite (Bothell, DC, USA) ultrasound machine with a high-resolution linear-array probe (using a 6–13 MHz, 50 mm linear probe). Grey-scale and Doppler examinations were performed while the patient was sitting, as many elderly patients were unable to stand for the entire examination. All images were anonymized. The ultrasound (US) report included: assessment of the patency of the common femoral vein (CFV), femoral vein (FV), popliteal vein (POPV), and deep calf veins. Concerning the superficial venous lower limb system, the great saphenous vein (GSV), small saphenous vein (SSV), the sapheno-femoral (SFJ) and sapheno-popliteal junction (SPJ), along with the main thigh tributary veins and existing perforators, were examined. The Valsalva maneuver was performed to assess venous reflux, with cut-off values of 1 s for the SFJ and 0.5 s for the SPJ.

The following features of the superficial inguinal lymph nodes were assessed: long axis measurement, shape (oval, round), echogenicity (normal, loss of echogenic hilum), and perifocal hypodermal oedema (defined as hyperechogenicity of the surrounding fat). With the patient in decubitus position, the infrainguinal lymph node varicose dilatation of the veins were graded as [17]: grade I (mildly dilated veins in the hilum, with visible echogenic hilum); grade II (moderately dilated veins filling the hilum, the normal echogenic hilum is absent, spontaneously recognizable lymph node), grade III (markedly dilated veins with abnormal morphology; normal lymph node features apparent only after compression maneuver and emptying of the varices). In cases where varicose veins were present in both limbs, the patient was assigned the most severe grade. The results were reported per patient rather than per limb to maintain adequate statistical power given the relatively small patient cohort. Each patient was assigned an identifier (ID), and when both legs were measured, a single ID was used for both measurements.

For the Shear Wave Elastography (SWE) the Advanced Breast mode was selected to provide measurements. A region of interest (ROI) of 3 mm was selected and placed in the lymph node sinus. Minimal transducer compression was used to empty the varices, and stability was assessed on the Stability Map until normal lymph node anatomy became apparent. Five elastographic measurements were obtained at different locations within the sinus for each lymph node, yielding a final mean value because the data were normally distributed. The values were expressed in kilopascals (kPa).

### 2.4. Statistical Analysis

Statistical analysis was conducted with Jamovi (version 2.6.44). The distribution and characteristics of the data were evaluated to select the appropriate statistical tests for each variable. The normality of quantitative variables was assessed using the Shapiro–Wilk test, supplemented by visual inspection of histograms and Q-Q plots.

For comparisons between two independent groups, the independent samples *t*-test was used when the assumption of equal variances (verified through Levene’s test) was met. If variances were unequal, Welch’s *t*-test was applied. In cases where variables are not normally distributed, the Mann–Whitney U test was employed.

Associations between categorical variables were analyzed using the Chi-square (χ^2^) test, provided that all assumptions were satisfied (specifically, expected frequencies ≥ 5 in at least 80% of the contingency table cells). Fisher’s exact test was used instead when these conditions were not met. The Haldane–Anscombe correction was used to prevent division-by-zero errors in contingency tables, to compute more accurate odds ratios, and to perform more precise Chi-square tests.

Linear regression analyses ensured that all underlying assumptions (linearity, independence, homoscedasticity, and normality of the residuals) were verified and respected.

All statistical tests were two-tailed, with a significance threshold set at *p* < 0.05. Results for normally distributed continuous variables are presented as mean ± standard deviation (SD), while non-parametric data are reported as median and interquartile range (IQR). Categorical variables are expressed as frequencies and percentages.

Data visualization was conducted using the Survey Plots module, the basic plotting module of the Jamovi app, and the ggplot2 add-on. These tools generated graphical representations of the analyzed variables, including stacked bar charts and error bar plots.

Phi coefficients and Cramer’s V were applied as appropriate to analyze associations between categorical variables.

Effect size for non-parametric comparisons was described using the rank-biserial correlation, and for parametric comparisons using Cohen’s D.

A robust mixed-effects model was used to examine the relationship between SWE and explanatory variables, including nodal varice grade, lymph node diameter, and other factors. The model incorporated a random effect for patient ID to account for intra-patient correlations, as multiple observations were made for the same patient (e.g., measurements from both lower limbs).

## 3. Results

**Figure 1:**
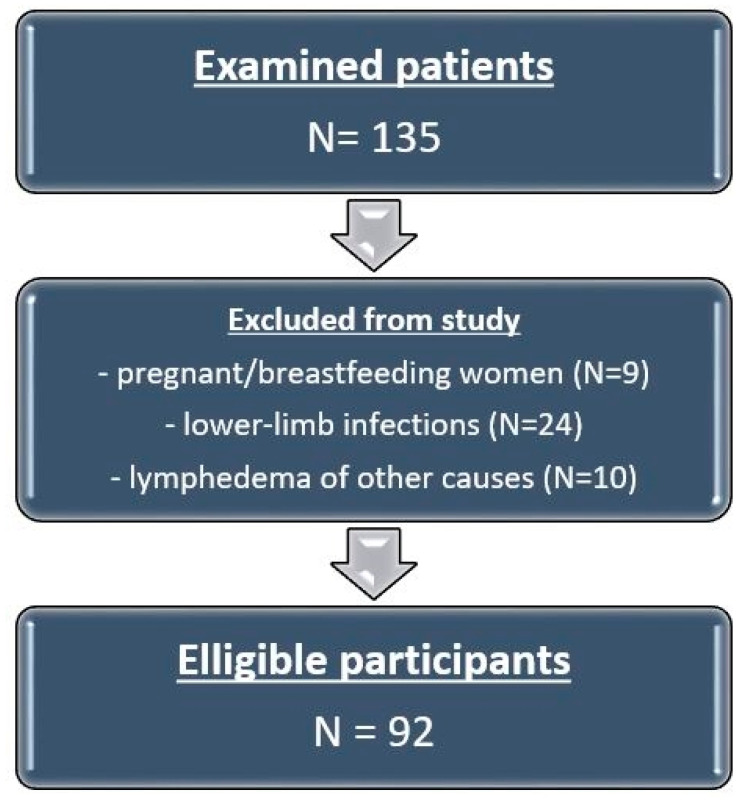
Standards for Reporting Diagnostic Accuracy Studies (STARD) flow diagram of patient inclusion.

Patient selection is depicted in Figure 1.

Ninety-two subjects were included in the study (69 women and 23 men; age range = 16–80 years).

Patients were classified according to the CEAP classification into incipient stages (C1–C2) and more advanced stages (C3–C6). Patients were placed in the more advanced C stage according to the most severe alterations in either one of the inferior limbs, even if they were asymmetrical.

Of the 92 patients examined, 69 (75%) were women, aged 29–88. Considering the CVD stages, grouped by severity, 15 (21.7%) of the female patients were in the incipient stages (C1–C2), and 54 (78.3%) were in more advanced stages (C3–C6), of whom 18 (33.3%) had healed or active ulcers (C5–C6). Among the 69 female patients, intranodal varices were identified in 33 (47.8%).

The male group ranged in age from 31 to 89. Of the 23 male patients, 2 (8.7%) were in the incipient stage (C1–C2) and 21 (91.3%) in the advanced stages (C3–C6), of whom 5 (23.8%) had healed or active ulcers (C5–C6). Of the 23 male patients, 7 (30.4%) had intranodal varices.

The main demographic characteristics, stratified by CEAP groups, are presented in Table 1.

Fifty percent of patients with CVD in their family history had intranodal varices, compared with only 36.4% of those with family history but without lymph node varices. Chi-square test did not identify a statistically significant difference between the two groups (χ^2^ = 1.74, *p* = 0.188). Odds Ratio (OR) of 1.75 [with a 95% confidence interval between 0.759–4.03], suggests a positive, but statistically insignificant, association between the two variables.

**Figure 2:**
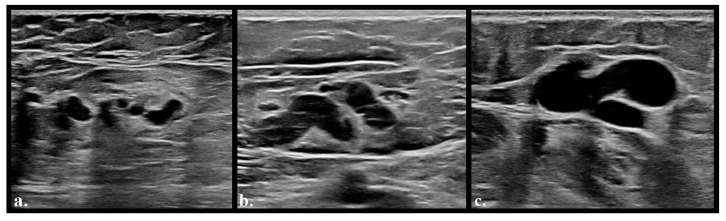
Lymph node varicose veins, from left to right: (**a**)—grade 1, (**b**)—grade 2, (**c**)—grade 3.

Of the 92 patients, 40 had intranodal varicose veins of varying degrees. Of these, 15 patients had bilateral intraganglionic varicose veins, while the rest had unilateral varicose veins. Of the 40 patients, twenty had intranodal varicose veins on an operated limb, while the rest had de novo varicose veins. Based on lymph node varicose vein grading, 19 patients were classified as grade I, 12 as grade II, and 9 as grade III. Figure 2 exemplifies the original classification of intranodal varices [16].

In patients without operated varicose veins, 33.8% had lymph node varicose veins, compared with 83.3% of patients with operated CVD. The chi-square test identified a significant difference (*p* < 0.001). The Odds Ratio demonstrated a 9.8 [with a 95% confidence interval of 2.59–37.1] greater likelihood that patients with operated varices would have intranodal varices; the Phi coefficient = 0.397 (moderate association).

Of the patients who underwent surgery for CVD, 99.3% of those with recurrences had intranodal varices, compared to only 33.8% of non-recurrent patients. Statistical findings showed a Chi-Square *p*-value < 0.001 and an odds ratio of 27.5 (95% confidence interval: 3.42–220), indicating a moderate association, with a Phi coefficient of 0.444.

No statistically significant differences were found between the two groups (with and without lymph node varicose veins) in the prevalence of significant saphenous reflux (*p* = 0.755) or cutaneous anomalies (*p* = 0.151).

**Figure 3:**
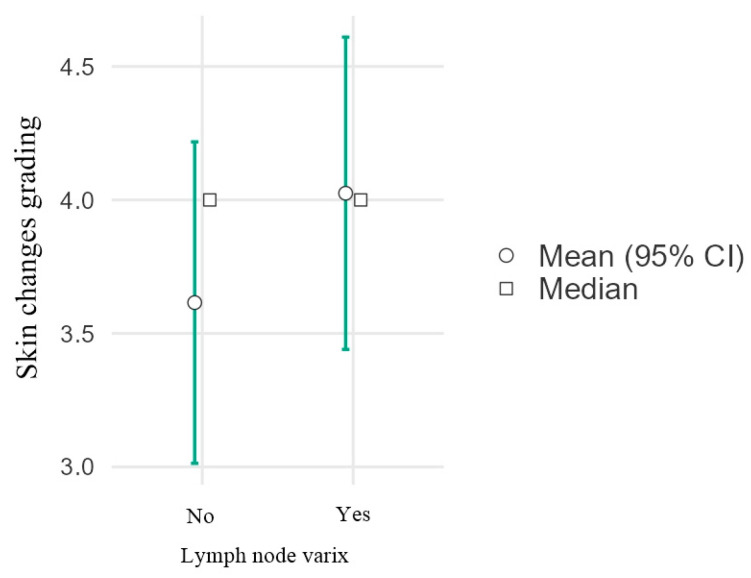
Error bar plot showing the correlation of skin changes grading (according to the CEAP classification) with the presence or absence of intranodal varices.

Figure 3 compares the grading of skin changes between patients without lymph node varix (“No”) and those with lymph node varix (“Yes”). The mean score is similar across the two groups, at approximately 4 in each. The median values are also very close to the respective means. The 95% confidence intervals are wide and overlap substantially, indicating variability in the data and suggesting no statistically significant difference in skin changes between the groups. Fifty percent of patients with trophic changes had at least grade 3 trophic changes, regardless of the presence of nodal varices. No significant difference in skin change score was observed between patients with and without lymph node varix (χ^2^(1) = 2.02, *p* = 0.155; OR = 2.35).

**Figure 4:**
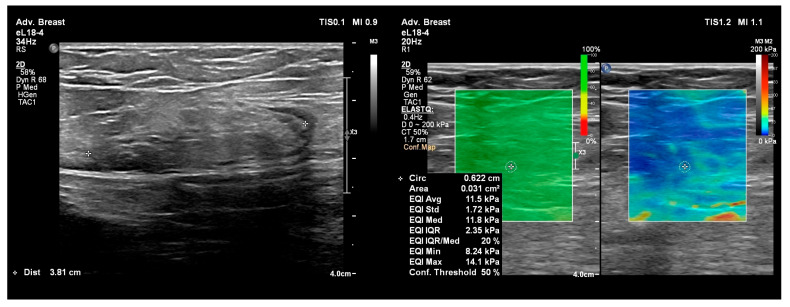
(**left**) image—long axis measurement of a groin lymph node; (**right**) image—Shear Wave Elastography (SWE) measurements in the same lymph node. A region of interest (ROI) of 3 mm was selected and placed in the lymph node sinus. Transducer compression was used to empty the varices, and the stability was assessed on the Stability Map.

**Figure 5:**
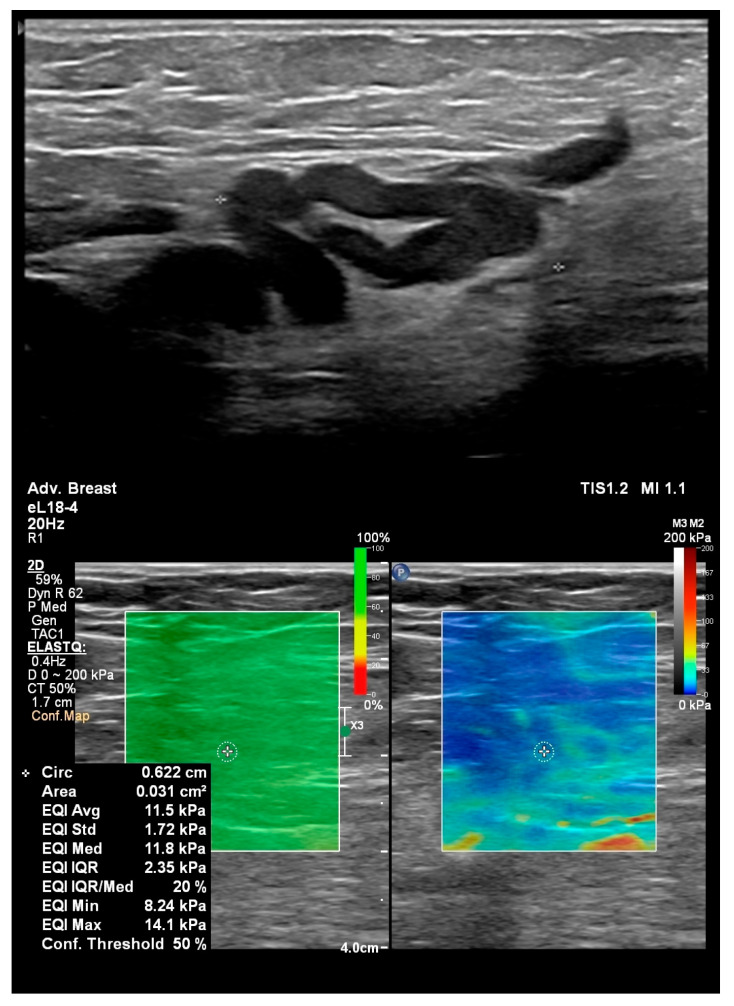
60-year-old female patient with post-surgical recurrence at the saphenofemoral junction. (**upper**) image—grade 3 varicose veins in the groin lymph node; (**lower**) image—SWE measurements in the same lymph node. A region of interest of 3 mm was selected and placed in the lymph node sinus. Transducer compression was used to empty the varices, along with assessment of stability on the Stability Map. The (**upper**) image and Supplementary Video S1 illustrate the trans-nodal veins and their connection to the great saphenous vein.

Figure 4 and Figure 5 illustrate lymph node long axis and SWE measurements. Supplementary Videos S1 and S2 show intranodal varices and their connections to the superficial venous system in the groin.

Table 2 presents Student’s *t*-test for equal variances, considering lymph node diameter between the two groups of patients (with and without intranodal varices). Patients with lymph node varices had a median of the maximum diameter of 30.9 mm (*p* = 0.052)

**Figure 6:**
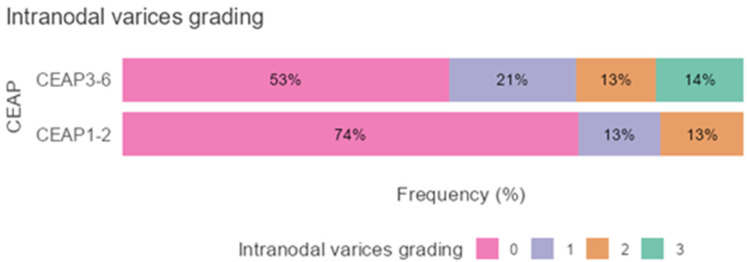
Stacked bar graph survey plots depicting frequency of intranodal varices grading in the CEAP categories (0—absent; 1—grade 1; 2—grade 2, 3—grade 3).

In Figure 6, intranodal varices were studied as an ordinal variable, based on their frequency within the CEAP categories. No statistically significant differences were found (Mann–Whitney *p* = 0.12)

**Figure 7:**
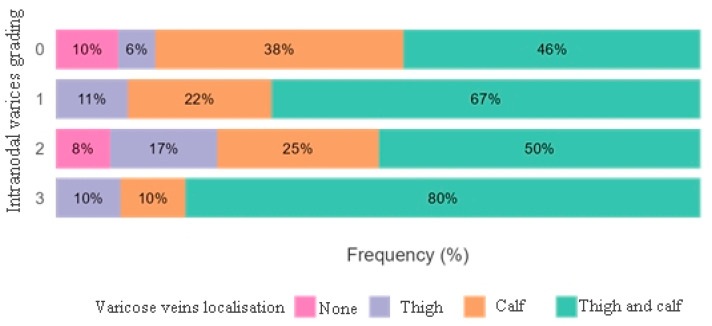
Stacked bar graph survey plots depicting the correlation between intranodal varices grading and varicose veins localisation.

In Figure 7, we assessed the correlation between intranodal varices and the grading of varicose vein localization. Although it did not demonstrate statistical significance (*p* = 0.077), grade 3 nodal varices were more frequently associated with varicose veins in both the thigh and calf.

Table 3 presents a correlation between the great saphenous vein diameter at the sapheno-femoral junction and the presence of nodal varix. The Mann–Whitney U test indicates a significant difference between the two groups (*p* = 0.036), and the rank biserial correlation indicates a small-to-moderate inverse association between ranks.

**Figure 8:**
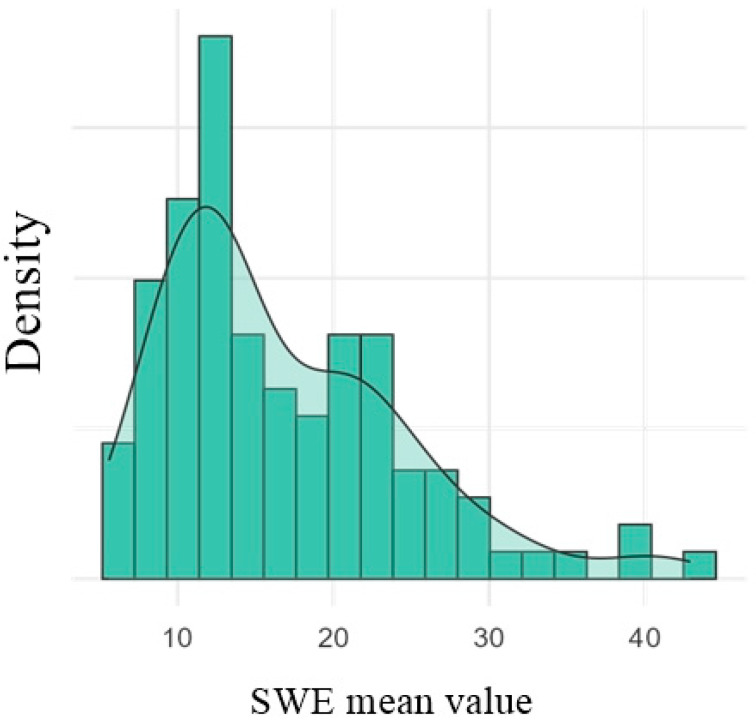
Histogram of SWE (Shear Wave Elastography), mean value of the groin lymph nodes.

The histogram of SWE mean values is depicted in Figure 8.

For the association between perinodal edema and varicose lymph nodes, the chi-square statistic was 3.24 (df = 1, *p* = 0.072). Although the *p*-value indicates a trend toward significance, the result does not exceed the significance threshold (*p* < 0.05, OR = 2.2). Most lymph nodes were oval (90), and only four were round. The echogenic hilum was preserved in all cases except those with grade II and III intranodal varix (21 patients).

**Figure 9:**
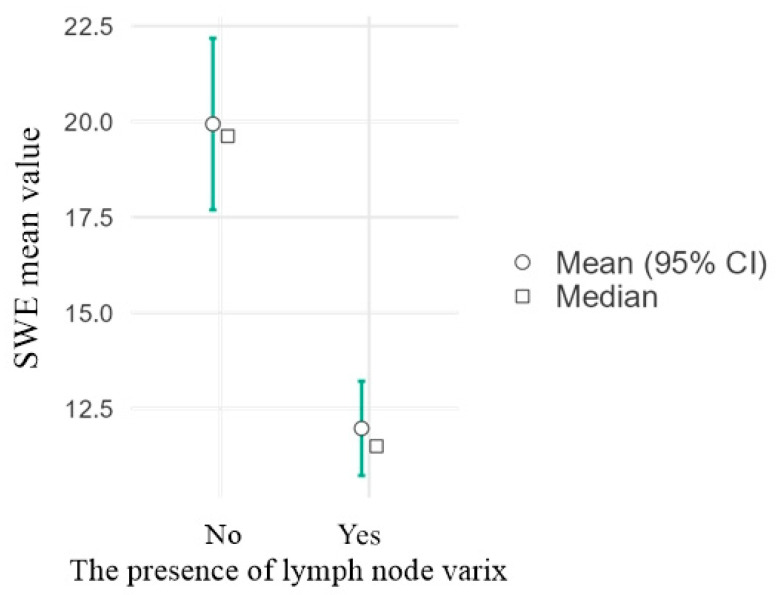
Error bar plot showing the correlation between SWE mean values and the presence or absence of intranodal varices.

Figure 9 shows the relationship between the mean lymph node SWE and the presence of lymph node varicose veins.

The SWE values were significantly higher in the group without lymph node varix compared to the group in which varix was present (20.1 ± 2.3 kPa vs. 12.2 ± 1.1 kPa; *p* < 0.05). The 95% confidence intervals did not overlap between the two groups.

Table 4 presents a correlation matrix of SWE with other essential variables.

In Table 5, a regression model was used to explain mean SWE values, accounting for lymph node varix grading and lymph node diameter.

To explore the association between the presence of other diseases (arterial hypertension and diabetes) and CEAP clinical stage (divided into two subgroups, CEAP 1–2 and CEAP 3–6), a contingency table was constructed and analyzed using a Chi-square test with Yates’ continuity correction. The chi-square test with Yates’ correction yields χ^2^(1) = 8.24, *p* = 0.004. Among patients without other diseases (*n* = 56), 76.8% were in CEAP stages C3–6, and 23.2% were in stages C1–2. Among patients with comorbidities (*n* = 51), 96.1% were in CEAP C3–6, and only 3.9% were in CEAP C1–2. The result is statistically significant, indicating a clear association between comorbidities and CEAP stage.

## 4. Discussion

In a previous study [16] published by our group, which employed a similar design but focused solely on LNVN description, we found a prevalence of 36.6% of lymph node varicose veins. In the current study, the prevalence was 43.4%. To the extent of our knowledge, the only other recent study in the literature with a similar design is by Liu et al. [18], who investigated the anatomy and haemodynamics of LNVN in patients with primary chronic venous disease, revealing an estimated prevalence of 12%. We might attribute the high prevalence to patient selection, as we included patients referred from the Dermatology and General/Vascular Surgery Departments who underwent thorough clinical investigation for CVD or presented for post-surgical recurrence.

In our study, the presence of other diseases (arterial hypertension and diabetes) was significantly associated with CEAP clinical stage (χ^2^(1) = 8.24, *p* = 0.004). The results support a significant association between comorbidities and the severity of chronic venous disease. Patients presenting with other conditions are significantly more likely to be in advanced stages (CEAP C3–6). This finding has significant implications for integrated clinical management, suggesting that assessing comorbidities should be an essential component of the treatment strategy. These results were supported by a study by Jarosikova et al. [19], which found a significant association between diabetes mellitus and CVD, as diabetes is characterized by a hypercoagulable state that increases the risk of deep vein thrombosis. It is also characterized by a pro-inflammatory response; consequently, it may exacerbate local inflammation in venous walls and accelerate the progression of venous alterations [18].

In a previous study [16], we found a moderate-to-high positive correlation between intranodal varices and the stage of chronic venous disease. In this study, nodal varices were examined as an ordinal variable, based on their frequency within the CEAP categories. It should be noted, however, that even though no statistically significant differences are found, the CEAP C3-C6 group is the only one with advanced (grade 3) intranodal varicose veins. We attribute the lack of statistical significance to the smaller sample size.

More than 83% of patients with operated CVD had lymph node varicose veins (*p* < 0.001; OR = 9.8 [95% CI 2.59–37.1]), with a Phi coefficient of 0.397 indicating a moderate association. In this group, in 99.3% of cases, we found recurrences via intranodal varices (*p* < 0.001, OR 27.5 [3.42–220], moderate association, Coeff Phi = 0.444). Although LNVN were found in subjects without CVD, as demonstrated by Uhl et al. [11], they were more frequent in post-surgical recurrence in our studies (present and [17]), as well as in other studies [12,14]. One could hypothesize its existence as an adaptive response to surgery, thus leading to CVD recurrence in the groin.

Ultrasound identification of intraganglionic varicose veins has the potential to be a major paradigm-shifting idea. Historically, inguinal recurrence was explained by postoperative neovascularization, incomplete ligatures, and pelvic reflux. Modern anatomy, although evaluated by few studies to date, suggests that this “neovascularization” may in fact represent pre-existing ganglionic venous networks that have become incompetent [11]. From a hemodynamic point of view, through this reflux pattern (femoral vein > ganglion vein > anterior accessory great saphenous vein (AAGSV)/other tributaries > typical varicose veins on the thigh), the lymph node becomes a shunt point (which we can consider an inguinal leak point) or a source of reflux independent of the saphenous trunk. This aspect is important in therapeutic decision-making because the standard protocol (GSV stripping, high ligation of the SFJ, phlebectomies [20,21]) may fail if the true source of reflux is LNVN, and GSV is merely a secondary vein of disturbance. If this aspect is not known preoperatively, the result will be a temporary disappearance of varicose veins, with unexplained recurrence [22]. The presence of intranodal varicose veins should change preoperative mapping, and the Doppler description should focus on the inguinal lymph nodes and their connections to the thigh’s tributaries, including groin reflux mapping, not just GSV mapping. The choice of therapeutic target shifts from saphenous trunk ablation to treatment of the dominant leak point. The literature already suggests, albeit indirectly, that reflux through LNVN is a relative contraindication to repeated inguinal surgical approaches [11]. Repeated dissection of this area produces lymphatic trauma, vascular remodeling, and aggravation of the network. The study may have major implications by introducing the concept of groin leak point disease, with the lympho-ganglionic venous reflux. However, the present study was not designed to evaluate surgical outcomes, recurrence rates, or rates of reintervention. At present, no specific surgical guidelines address the management of intraganglionic varicose veins, and therefore, our findings should be interpreted as hypothesis-generating rather than practice-changing. Recognizing these venous networks during preoperative duplex examination may improve anatomical understanding of inguinal reflux patterns and could help recognize certain cases of unexplained recurrence described in the literature. Future prospective outcome-based studies, including standardized follow-up and comparative analyses of different treatment strategies, are necessary to determine whether targeted management of intraganglionic reflux improves clinical outcomes.

Patients with lymph node varix had a median maximum lymph node diameter of 30.9 mm (*p* = 0.052). Although not statistically significant in our study, this finding was supported by Liu et al. [18], who reported that lymph nodes tend to be larger. In this study, LNVNs exhibited variability in shape, size, vascularity, and echogenicity, with no significant differences between the competent and incompetent groups, except for the presence of reflux phenomena. Reflux within LNVNs often involves connections to the great saphenous vein and anterior accessory GSV. The study identified LNVN diameters ranging from 0.8 mm to 10.8 mm. Instead of measuring diameters, we classified lymph node varix according to our original classification, as previously presented in another study [16].

The histogram of SWE mean values (Figure 4) indicates a right-skewed distribution, characterized by a significant concentration of data in the 10–20 kPa range. This distribution suggests that most lymph nodes examined exhibit low-to-moderate tissue stiffness. A small number of elevated values (>30 kPa) indicate the presence of a subset of lymph nodes with increased stiffness, possibly associated with pathologic changes that we did not account for and were previously unknown to us.

We analyzed the relationship between peri-nodal edema and the presence of varicose lymph nodes. The odds ratio value of 2.2 indicates that the presence of edema is associated with more than twice the odds of lymph node varix compared to its absence. The OR suggests a potential relationship that warrants further investigation. Further data collection is recommended to confirm these preliminary observations.

No significant difference in skin change score was observed between patients with and without lymph node varix (χ^2^(1) = 2.02, *p* = 0.155; OR—2.35), suggesting that the presence of nodal varices does not significantly influence the associated clinical tegumentary appearance. Although not statistically significant, the odds ratio of 2.35 indicates that patients with lymph node varices are 2.35 times more likely to exhibit trophic changes than those without them.

The presence of lymph node varix was associated with a significant reduction in tissue stiffness, as measured by SWE (12.2 ± 1.1 kPa vs. 20.1 ± 2.3 kPa; *p* < 0.05). This may reflect structural and hemodynamic changes in the lymph nodes, characterized by increased laxity of the lymph node parenchyma. These results suggest that SWE assessment, combined with identification of lymph node varices, could improve lymph node characterization in imaging evaluations. In the context of lymph nodes, vascular abnormalities, such as intranodal varix, may influence SWE measurements by altering tissue mechanical properties. Our findings demonstrate that lymph nodes with nodal varices exhibit significantly lower SWE values, which may be attributed to increased vascularization and reduced tissue density. As shown in Table 4, elastographic values correlate with lymph node diameter (*p* = 0.039; weak negative correlation) and varicose vein grade (*p* < 0.001; moderate negative correlation). Precompression alters the prestress state of the tissue: a slightly compressed tissue becomes more rigid due to the nonlinear behavior of soft materials (an apparent increase in elastic modulus). This alters the shear wave velocity, typically resulting in higher values with increasing precompression [23].

Reviews and papers on lymph node elastography note that the technique is operator-dependent and emphasize the need to minimize and standardize precompression to obtain reproducible and comparable values [24]. In this study, a systematic technique was employed to reduce precompression, which involved maintaining light contact with the skin, applying sufficient gel, using the precompression indicator (which provides visual feedback and a color bar indicating compression quality), and keeping the ROI size constant.

As shown in Table 5, if the varicose vein grade increases by one unit, the SWE mean value decreases by 3.27 (*p* < 0.001). A significant negative association was found between lymph node diameter and SWE; each 1-unit increase in lymph node diameter was associated with a 0.185-unit decrease in SWE (*p* = 0.025). The regression model accounts for 27.1% of the variability in SWE values. Although the lymph node varix in post-surgical recurrence cases appeared to be a potentially critical factor influencing SWE values, no statistically significant association was found in this study (Estimate = −1.53, *p* = 0.444). Further investigation with a larger sample size or alternative methodologies may be necessary to fully understand its potential impact. There are variations across devices and techniques; the exact quantitative relationship between the magnitude of precompression and the kPa increase in lymph node pressure is not consistently defined across devices, as many studies focus on the thyroid, breast, or salivary glands [25] or on experimental studies [26]. Extrapolation to lymph nodes is based on mechanistic evidence and only a few dedicated clinical studies [27].

Our study has a few limitations. Firstly, it included a relatively small sample size, 92 participants, which may limit the statistical power and generalizability of the results, especially for subgroup analyses (operated vs. non-operated patients). The cross-sectional observational design of the study does not allow the establishment of a causal relationship between the presence of intranodal varices and the progression of chronic venous disease, but only highlights associations. Also, the predominance of women (≈75%) may influence the extrapolation of the results to the general population with CVD. Another limitation is the lack of a healthy control group. The comparison was made between patients with different forms of CVD, without including a population without venous disease, which limits the interpretation of elastographic changes as specific to the pathology. We used an original classification of the intranodal varicose veins; although useful exploratively, it is not yet externally validated, which may affect the reproducibility of results between centers. Doppler ultrasound and shear-wave elastography are operator-dependent methods, and the lack of assessment of intra- and inter-observer variability may affect measurement accuracy. This study does not include longitudinal follow-up, so the role of intranodal varices as a true marker of CVD progression remains to be confirmed in larger cohort studies.

## 5. Conclusions

Intranodal varices may be regarded as potential markers of disease progression, as the presence of lymph node varicose veins correlates with increased disease severity. Vascular abnormalities, such as intranodal varices, affect SWE measurements, presumably by altering tissue mechanical properties. These venous dilations within the groin lymph nodes can be detected via ultrasound. Recognizing lymph node varicose veins could enhance diagnostic accuracy and inform treatment planning, underscoring the importance of considering alternative pathways of venous recurrence and their impact on CVD management.

## Figures and Tables

**Table 1:** Descriptive statistics by CEAP classification.

	Patients (%)	CEAP 1–2	CEAP 3–6	OR [95% IC]	Test	*p*
Sex						
M	23 (25%)	2	21	2.43 [0.50–11.7]	FET	0.341
F	69 (75%)	13	56	0.41 [0.51–11.7]	FET	0.341
Environment (Urban)	66 (71.3%)	12	53	0.587 [0.15–2.28]	FET	0.543
Obesity BMI > 30	26 (26.1%)	2	24	2.94 [0.61–14.01]	FET	0.218
Family history	48 (52.1%)	8	40	1.06 [0.349–3.2]	CHI	0.92
Surgical intervention for CVD	18 (19.5%)	0	18	9.6 * [0.55–148]	FET	**0.037**
Post-surgical recurrence	15 (16.3%)	0	15	7.69 * [0.44–135]	FET	0.119
Arterial hypertension	38 (41.3%)	2	36	5.71 [1.2–27]	CHI	**0.016**
Diabetes mellitus	17 (18.4%)	0	17	8.97 * [0.49–148]	FET	0.064

* Haldane-Anscombe Correction. CHI—Chi-square Test. FET—Fisher’s Exact Test. Bolded values in *p* column are statistically significant (*p* < 0.05).

**Table 2:** Student’s *t*-test for equal variances between the two patient groups.

Group Descriptives						
	Group	N	Mean	Median	SD	SE
Lymph node diameter	Without nodal varix	52	27.3	26.5	8.15	1.13
	With nodal varix	40	30.9	30.0	9.35	1.48

Definition of abbreviations: N—number of cases; SD—standard deviation; SE—standard error.

**Table 3:** Student’s *t*-test for independent samples.

Independent Samples *t*-Test							
		Statistic	*p*	Mean difference	SE difference		Effect Size
GSV * Diameter	Mann–Whitney U	1056	0.036	1.5		Rank biserial correlation	−0.238
Group Descriptives							
	Group	N	Mean	Median	SD	SE	
GSV * Diameter	0	63	6.57	6	2.83	0.356	
	1	44	4.9	5	3.8	0.573	

Note. H_a_ μ0 ≠ μ1, * GSV—Great Saphenous Vein, SD = standard deviation, SE—Standard Error. The positive skewness of the distribution indicates that the data are not normally distributed. The Shapiro–Wilk test statistic was <0.001, indicating that the mean SWE values were not normally distributed. Nonparametric tests were used.

**Table 4:** Correlation matrix between elastographic values and other variables.

Correlation Matrix		SWE Value (Mean)
GSV Diameter (mm)	Spearman’s rho	0.07
	Df	90
	*p*-value	0.505
GSV reflux (s)	Spearman’s rho	−0.063
	Df	90
	*p*-value	0.550
SSV diameter (mm)	Spearman’s rho	0.024
	Df	90
	*p*-value	0.818
SSV reflux (s)	Spearman’s rho	−0.05
	Df	90
	*p*-value	0.637
Lymph node diameter (mm)	Spearman’s rho	−0.215
	Df	90
	*p*-value	**0.039**
Lymph node varix grading	Spearman’s rho	−0.541
	Df	90
	*p*-value	<**0.001**

Definition of abbreviations: GSV = great saphenous vein, SSV = small saphenous vein, mm = millimeters, s = seconds, SWE = Shear Wave Elastography. Bolded values in the SWE Value column are statistically significant (*p* < 0.05).

**Table 5:** Regression model for SWE mean values, by lymph node varix grade and lymph node diameter.

Model Coefficients—SWE Value (Mean)				
Predictor	Estimate	SE	*t*	*p*
Intercept	24.372	2.4132	10.10	<0.001
Lymph node varix grade	−3.271	0.6862	−4.77	<0.001
Lymph node diameter	−0.185	0.0814	−2.27	0.025

Definition of abbreviations: SE—standard error, SWE = Shear Wave Elastography.

## Data Availability

Data is unavailable due to privacy or ethical restrictions.

## Supplementary Materials

The following supporting information can be downloaded at: https://www.mdpi.com/article/10.3390/diagnostics16060905/s1, Video S1: 60-year-old female patient with post-surgical recurrence at the saphenofemoral junction. Upper image—grade 3 varicose veins in the groin lymph node; lower image—SWE measurements in the same lymph node. A region of interest of 3 mm was selected and placed in the lymph node sinus. Transducer compression was used to empty the varices, along with assessment of stability on the Stability Map. The upper image and Video S1 illustrate the trans-nodal veins and their connection to the great saphenous vein; Video S2: 65-year-old man with C3 disease with primary non-operated CVD; grade 2 lymph node varicose veins, connected to the inferior epigastric vein and the saphenous vein.

## Abbreviations

The following abbreviations are used in this manuscript:

CVD	Chronic venous disease
SWE	Shear wave elastography
US	Ultrasonography
CEAP	Clinical, Etiology, Anatomic, and Pathophysiology classification
CVI	Chronic venous insufficiency
BMI	Body mass index
LNVN	Lymph node venous network
CFV	Common femoral vein
FV	Femoral vein
POPV	Popliteal vein
GSV	Great saphenous vein
SSV	Small saphenous vein
SFJ	Sapheno-femoral junction
SPJ	Sapheno-popliteal junction
ROI	Region of interest
kPa	KiloPascals
ID	Identifier
SD	Standard deviation
IQR	Interquartile range
STARD	Standards for Reporting Diagnostic Accuracy Studies
OR	Odds Ratio
